# Time trends in spine surgery in Italy: a nationwide, population-based study of 1,560,969 records of administrative health data from 2001 to 2019

**DOI:** 10.2340/17453674.2025.43188

**Published:** 2025-03-13

**Authors:** Marina TORRE, Andrea PIAZZOLLA, Enrico CIMINELLO, Tiziana FALCONE, Eugenio CARRANI, Simona PASCUCCI, Michela FRANZÒ, Giuseppe BARBAGALLO, Vincenzo VITIELLO, Gustavo ZANOLI, Alessia BIONDI, Letizia SAMPAOLO, Veronica MARI, Francesco LANGELLA, Pedro BERJANO

**Affiliations:** 1 Italian National Institute of Health, Rome; 2 UOSD Spinal Deformity Center, AOU Consorziale “Policlinico”, Bari; 3 Department of Mechanical and Aerospace Engineering, “Sapienza” University of Rome, Rome; 4 Department of Neurosurgery, University of Catania, Catania; 5 Department of Neurosurgery, San Giovanni Bosco Hospital, Naples; 6 Department of Orthopedics, Casa di Cura Santa Maria Maddalena, Occhiobello (RO); 7 Ospedale Galeazzi-Sant’Ambrogio, Milan, Italy

## Abstract

**Background and purpose:**

The use of spinal implants has increased substantially. Their widespread use raises public health concerns. We aimed to study spinal surgery trends in Italy from 2001 to 2019 and present a mapping for ICD9-CM codes potentially related to spinal diagnoses and procedures.

**Methods:**

ICD9-CM codes of interest were selected and mapped to clinically meaningful spinal diagnostic categories and procedure classes. The Italian National Hospital Discharge Records database was then browsed according to these codes. Surgical volumes and trends were described. Population incidence rates (IR) were estimated and provided with 95% confidence intervals (CI). Variations in IRs were reported in terms of incidence rate ratio. The statistical significance of counts and IR time series trends was assessed by using the Cox–Stuart test.

**Results:**

1,560,969 spinal procedures were extracted from 209,818,966 admissions registered nationally. The annual number of spinal procedures increased significantly by 67%, from 58,369 in 2001 to 97,636 in 2019 (P < 0.002). 1,040,326 (67%) procedures did not include implants, while 590,643 (33%) used implants, 395,450 (25%) associated with fusions and 125,193 (8%) with non-fusions. Population IRs increased from 100.9 (CI 100.1–101.7) to 163.2 (CI 162.2–164.3) episodes per 100,000 inhabitants. Surgical volumes for non-implant-related procedures remained stable, while implant-related procedures increased significantly, by 420% over the 19 observed years (P = 0.002).

**Conclusion:**

Spinal surgical procedures and their population incidence rates increased significantly. Fusions and other implant-related procedures increased substantially for most diagnostic categories. An ICD9-CM mapping for spinal diagnoses and procedures as a reproducible tool for further explorations was presented.

Surgical treatment of spinal conditions provides improvement of health-related quality of life or value in terms of quality-adjusted life-years (QALY) in several spine conditions and procedures [1-11]. Using implants during surgical procedures aims to enhance stability, modify alignment, or both. In the last decades, the use of spinal implants has increased substantially [12]. Although spinal implants are expected to improve surgical outcomes, their widespread use on a population basis may raise public health concerns.

While spinal implants are designed to improve patients’ quality of life, the procedures and the implant itself carry a risk, including potential adverse events leading to recalls [13]. Such events may have catastrophic consequences for patients and healthcare systems [14]. The incidence of procedures per 100,000 inhabitants per year, 1 of the 6 indicators defined by the Lancet Commission on Global Surgery, is a key measure for monitoring universal access to safe, affordable surgical and anesthesia care [15]. Therefore, studying time trends and incidence analyses is a crucial reference for public health decision-making.

We aimed to study spinal surgery trends in Italy over 19 years, from 2001 to 2019, using as a source the population-based data from the National Hospital Discharge Records (HDR) Database and present specific mapping from ICD9-CM coding relevant to clinically meaningful spinal diagnoses and procedures.

## Methods

### Study design

This study is designed as a retrospective population study based on HDR in accordance with the Strengthening the Reporting of Observational studies in Epidemiology (STROBE) [16].

### Italian demographics, national health system organization, and health data collection

Italy is administratively divided into 19 regions and 2 autonomous provinces. The population increased from 58 million at the beginning of 2001 to 60 million in 2019. The Italian National Health System (NHS) was established in 1978 based on 3 fundamental principles: assistance universality, access equality, and solidarity. Regions are responsible for health services planning and organization in a situation of autonomy within the framework of the 3 principles mentioned above. The NHS, funded nationally through general taxation, is organized by different public bodies cooperating to provide health care for all citizens.

Established in 1994, the Hospital Discharge Records (HDR) Database contains information concerning all national hospital admissions. The total HDR number has varied from 12,940,082 in 2001 to 8,537,262 in 2019, with a steadily decreasing trend related to decreased hospital admissions following policies adopted to improve their appropriateness. Hospitals are required to collect data using the Hospital Discharge Form (HDF) (“Scheda di Dimissione Ospedaliera”—SDO) for both ordinary and day-hospital admissions. On discharge, clinicians at all public and private hospitals fill in the HDF, including the patient’s demographics, information regarding the hospitalization, both administrative and clinical, such as main diagnosis, up to 5 concomitant diagnoses, main procedure, and secondary procedures (up to 5 until 2016, extended to 10 since 2017). Diagnoses and procedures are coded by using the International Classification of Diseases, 9th revision – Clinical Modification, ICD9-CM (version 2007) (https://www.cdc.gov/nchs/icd/icd9cm.htm; Italian version: http://www.salute.gov.it/imgs/C_17_pubblicazioni_2251_allegato.pdf). The hospitals transmit data to the Regional Health Authority, which forwards it to the Ministry of Health after consolidation. Through the HDFs, Regions can gather more information than the Ministry of Health requires. Each Region reimburses hospital admissions based on the main diagnosis and treatment reported in HDFs, summarizing the condition that required the hospital’s highest resource allocation for the episode.

Consequently, coverage (i.e., the ratio between the number of hospitals collecting HDF and the number of hospitals active in the country) is almost comprehensive. The Ministry of Health is responsible for checking the quality of the data collected and making every effort to address potential sources of bias. During the period considered by our study, coverage increased from 94.2% in 2001 to 99% in 2019 [17].

### Identification and categorization of diagnoses and procedures

A technical, multidisciplinary panel composed of an engineer, the head of the Italian implantable prostheses registry (MT), an information technologist (ECa), a statistician (ECi), 2 orthopedic spine surgeons (PB, AP), an orthopedic surgeon expert in evidence-based medicine and registries (GZ), and 2 neurosurgeons (VV, GB) was established to define the methodological principles of the future Italian Spine Registry (RIDIS), one of the registries included in the broader framework of the Italian Implantable Prostheses Registry (Registro nazionale delle Protesi Impiantabili, RIPI available at https://ripi.iss.it/ripi/en/) [18]. All the surgeons were designated by their national scientific societies. Analyzing the landscape of spinal procedures in the country was one of the topics assigned to the panel. To achieve this goal, the panel adopted a 2-step approach to select the diagnosis and procedure ICD9-CM codes to be used as a reference to browse the HDR national database.

First, a set of categories of diagnoses and a set of classes of procedures were defined a priori. For diagnoses, categories were sorted hierarchically following an etiological criterion, from that with the highest to that with the lowest impact on the patient’s health. For procedures, classes were hierarchically ordered based on being or not being spinal fusions and the likelihood of implanting or not implanting a device. As HDRs do not contain information regarding implanted devices and ICD9-CM codes for procedures do not always explicitly describe their use, the panel assumed that procedures could be classified into those with a low probability and those with a high probability of using implants, based on standard practice. For example, excision of the intervertebral disc was considered a procedure with a low probability of implant usage. In contrast, spinal fusions were considered procedures with a high probability of using implants.

Second, all the codes of the 2007 Italian version of the ICD9-CM manual for diagnoses (16,213 codes) and procedures (4,460 codes) were analyzed by 4 surgeons (PB, AP, VV, GB) who blind-selected those pertinent to spinal pathology based on their knowledge and expertise. For the diagnoses, all the codes selected by at least 1 of the surgeons were included in the final list. For the procedures, all the codes proposed by 3 or 4 surgeons were included in the final list, while the ones proposed by only 2 were discussed among the panel to reach a final consensus. Codes proposed by only 1 surgeon were excluded. The mapping of each code to only 1 diagnostic category or procedure class was discussed and finalized after consensus.

### HDR Database management, data extraction, and processing

The Italian National Institute of Health (ISS) receives from the Ministry of Health, every year, a subset of variables of the national HDR Database (Banca dati SDO) in CSV format. This dataset, including all the admissions performed at the national level in the last year, is harmonized by ISS with the datasets received in the previous years to get uniform variable names and modalities, imported and processed in a relational SQL database. For this study, 2 IT professionals (ECa and VM) queried the obtained database for the admissions performed from January 1, 2001, to December 31, 2019. They extracted all the records, including the selected diagnoses and procedures. The implemented query string had the following structure: “(each of the spine-related diagnosis codes, separated by OR Boolean operator) AND (each of the spine-related procedure codes, separated by OR Boolean operator).” This query strategy retrieved all records with at least 1 spine-related diagnosis and 1 spine-related procedure. Each record was then assigned to a diagnostic category; in the case of more than 1 etiology registered in the same record, the case was attributed to the category of the highest level in the defined hierarchy. Furthermore, each record was assigned to a procedure class; if more procedures did not belong to the same class and were registered in the same record, the case was attributed to the class with the highest probability of implant usage. For example, if the ICD9-CM code 03.1, “Division of intraspinal nerve root,” not involving implant use, and the code 81.0, “Spinal fusion,” likely involving implant use, were reported in the same record, the case was assigned to the implant-related procedure class.

### Statistics

Hospital admissions total caseload, its temporal trends, and incidence rates on the population by year from 2001 to 2019 were analyzed by diagnostic category and procedure class. Incidence rates (IR) per 100,000 inhabitants were estimated via Poisson model and provided with 95% confidence intervals (CI), while variations in IRs were reported by incidence rate ratio (IRR) and relative CI for comparability with other studies in international literature. Moreover, the statistical significance of the observed time series trends was also assessed by the Cox–Stuart test. This allows for checking variations either as actual trend changes over time, or random fluctuations around the underlying trend process. The significance threshold of the P value was fixed at 0.05. The statistical analysis was conducted by software R version 3.6.3 (2020-02-29)—“Holding the Windsock” (R Foundation for Statistical Computing, Vienna, Austria).

### Ethics, data sharing plan, funding, use of AI, and disclosures

HDRs are collected by the Ministry of Health in an administrative database and anonymized before their delivery to the Italian National Institute of Health, which uses them to perform epidemiological studies related to its public health mission. All the data is presented in aggregated form, and no Ethics Committee approval was needed under national law to conduct this study. Based on the large numbers used, the probability of identifying individuals who underwent the selected procedures was assumed to be extremely low. The study was conducted following the principles of the Declaration of Helsinki.

The data analyzed in this study is subject to the following licenses/restrictions: the analysis of the data used in this study complies with the European General Data Protection Regulation (EU GDPR 2016/679), which authorizes the processing of personal data relating to hospital discharge forms by ISS and other public institutions for reasons of public interest in public health. This study did not require written consent to participate, as per national legislation and institutional requirements. Requests to access these datasets should be directed to MT, marina.torre@iss.it. The participation of the authors Pedro Berjano and Francesco Langella from IRCCS Ospedale Galeazzi-Sant’Ambrogio in this study was supported and funded by the Italian Ministry of Health – “Ricerca Corrente.”

AI tools were not used for this submission.

The authors declare no competing interest. Complete disclosure of interest forms according to ICMJE are available on the article page, doi: 10.2340/17453674.2025.43188

## Results

**Figure 1:**
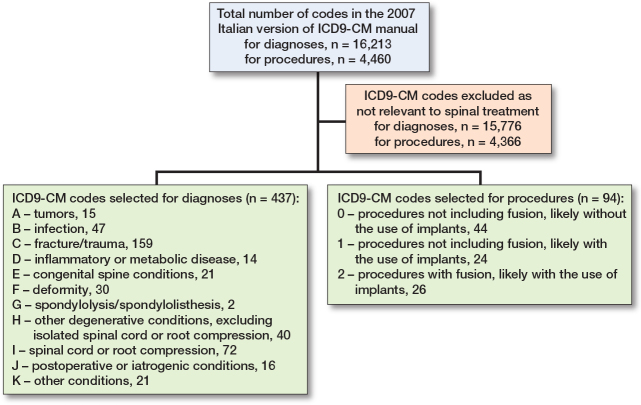
ICD9-CM codes selection.

11 diagnostic categories and 3 procedure classes of interest were identified. Diagnostic categories were hierarchically sorted from category A (Tumor), which has the highest patient health impact, to category K (Other conditions). In total, 437 diagnostic and 94 procedure codes were selected from 16,203 diagnoses and 4,460 procedures (Figure 1).

The full list of the selected codes and their complete mapping is available as Supplementary data (Table S1. Selected ICD9-CM diagnosis codes by diagnostic category; Table S2. Selected ICD9-CM procedure codes by procedure class).

**Figure 2:**
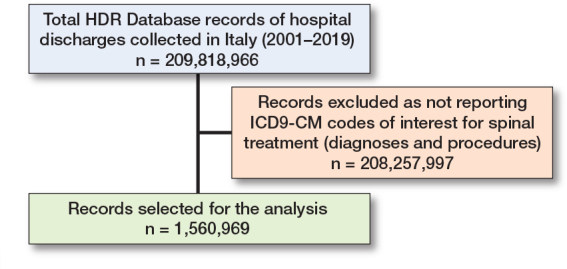
HDRs selection. See Table 2 for the record breakdown by diagnostic categories and procedure classes.

The HDR Database included 209,818,966 records of hospital discharges from 2001 to 2019 performed nationally. 1,560,969 HDRs pertinent to spinal treatments were extracted (Figure 2).

Table 1 shows the 11 diagnostic categories and the 3 procedure classes in hierarchical order, along with the corresponding number of assigned ICD9-CM codes and related records.

**Table 1:** Diagnostic categories and procedures classes: number of assigned ICD9-CM codes and records

Description		Number of codes	Number of records
Diagnostic categories			
A	Tumor	15	86,099
B	Infection	47	24,946
C	Fracture	159	179,389
D	Inflammatory or metabolic disease	14	16,247
E	Congenital spine conditions	21	37,277
F	Deformity	30	32,268
G	Spondylolysis/spondylolisthesis	2	48,599
H	Other degenerative conditions, excluding isolated spinal cord or root compression	40	234,601
I	Spinal cord or root compression	72	844,763
J	Postoperative or iatrogenic conditions	16	33,900
K	Other conditions	21	22,880
Total		437	1,560,969
Procedure classes			
0	Procedures not including fusion, likely without the use of implants	44	1,040,326
1	Procedures not including fusion, likely with the use of implants	24	125,193
2	Procedures with fusion, likely with the use of implants	26	395,450
Total		94	1,560,969

During the 19 years, 844,763 (54%) procedures were performed to treat spinal cord or nerve root compression (Table 2). Other degenerative conditions accounted for 234,601 procedures (15%), followed by fracture/trauma (179,389, 12%), and tumor (86,099, 5.5%). These 4 categories accounted for 1,344,852 (86%) cases. 1,165,519 procedures (75%) were not fusions and 1,040,326 (67%) likely did not include any implant, while 125,193 (7.9%) likely included implants. 395,450 (25%) were fusions likely with implants. Globally, 520,643 (33%) of procedures were related to the likely use of implants.

**Table 2:** Overall caseload by diagnosis category and procedure class (2001–2019)

Diagnostic category/description		Procedure class			Total (%)
		0	1	2	
		Non-fusion without implant	Non-fusion with implant	Fusion with implant	
A	Tumor	69,798	6,519	9,782	86,099 (5.5)
B	Infection	23,258	449	1,239	24,946 (1.6)
C	Fracture/trauma	27,478	91,710	60,201	179,389 (12)
D	Inflammatory or metabolic disease	14,810	201	1,236	16,247 (1.0)
E	Congenital spine condition	4,043	631	32,603	37,277 (2.4)
F	Deformity	5,433	532	26,303	32,268 (2.1)
G	Spondylolysis/spondylolisthesis	2,378	1,417	44,804	48,599 (3.1)
H	Other degenerative conditions	136,620	5,277	92,704	234,601 (15)
I	Spinal cord or root compression	714,033	15,786	114,944	844,763 (54)
J	Postoperative or iatrogenic conditions	28,710	2,019	3,171	33,900 (2.2)
K	Other conditions	13,765	652	8,463	22,880 (1.5)
	Total	1,040,326	125,193	395,450	1,560,969
	%	67	7.9	25	

From 2001 to 2019, the annual spinal procedures increased by 67% (P < 0.002), from 58,369 to 97,636 (Table S3, see Supplementary data). Considering diagnoses, procedures to treat tumor increased by 41% (P = 0.002), from 3,577 to 5,056, fracture/trauma by 285% (P = 0.002) from 3,658 to 14,092, deformity by 293% (P = 0.002), from 771 to 3,033, spondylolysis/spondylolisthesis by 368% (P = 0.002), from 1,022 to 4,782, and other degenerative conditions by 98% (P = 0.002), from 7,885 to 15,580. Procedures to treat spinal cord or nerve root decompression were 38,368 in 2001 and 45,240 in 2019 (P = 0.5). An overall increase in the implant-related procedures (P = 0.002 for both non-fusion and fusion with implant use) was observed; procedures related to fusion with implant use increased significantly for all diagnostic categories (P = 0.002), bar other conditions (K). For the non-implant-related procedures, the overall trend was decreasing (P = 0.09).

**Figure 3:**
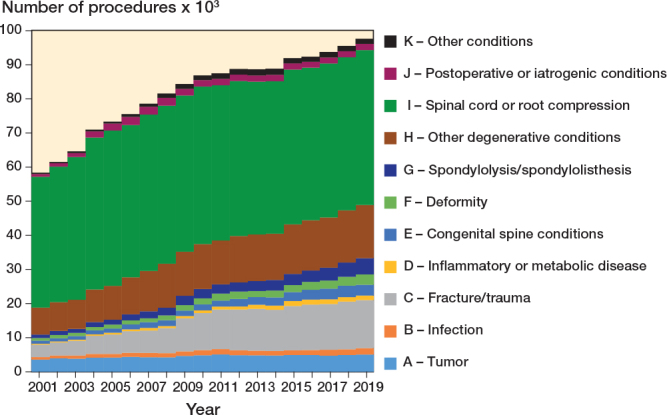
Time trends of procedures by diagnostic category (2001–2019).

**Figure 4:**
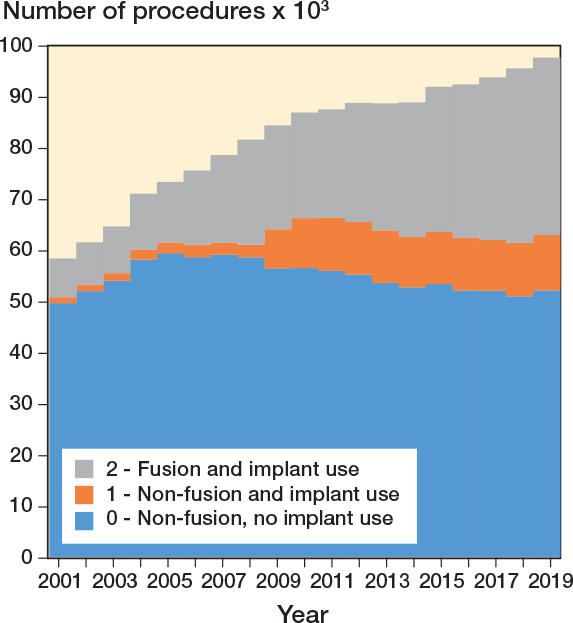
Time trends of procedures by procedure class (2001–2019).

Time trend by diagnostic category and procedure class showed that the overall number of spinal surgeries increased over the years (P < 0.001), mainly due to the treatment of trauma when looking at diagnoses (Figure 3) and to the use of implants when looking at procedures (Figure 4).

**Figure 5:**
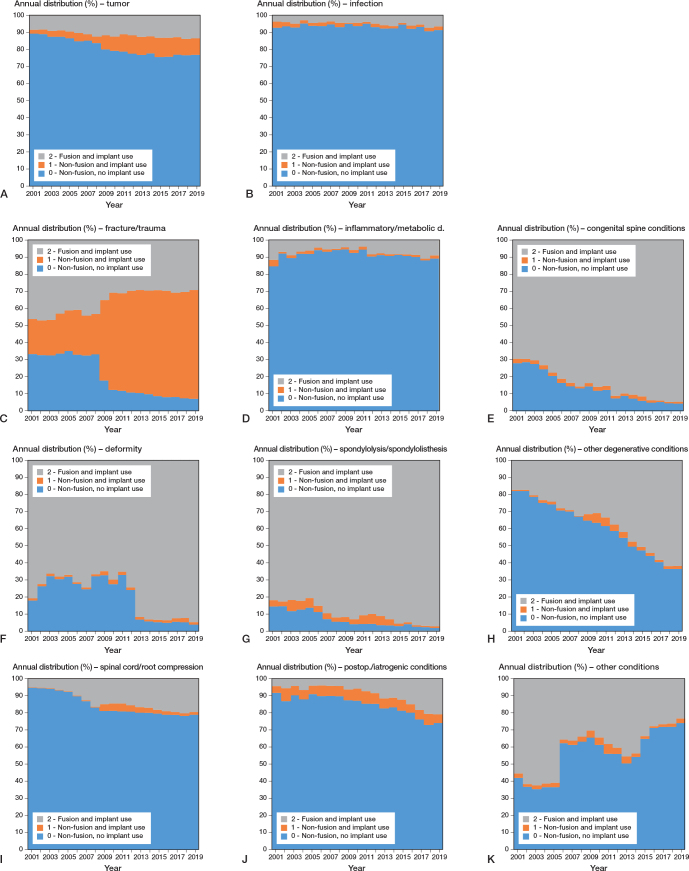
Time trends of relative frequencies of procedures for each diagnostic category (A–K) by procedure class (2001–2019).

Regarding frequencies of procedures for each diagnosis category by procedure class, change over time showed treatment of fracture/trauma (C) without implants was 33% in 2001 (1,211 cases out of 3,658) and decreased to 7% (969 cases out of 14,092) in 2019; non-fusions with implants tripled in the same period from 21% (756 cases out of 3,658) to 64% (9,000 cases out of 14,092) (Figure 5). For congenital spine conditions (E), deformity (F), and spondylolysis/spondylolisthesis (G), the use of implants, being already the preferred treatment in 2001, in 80% of cases (2,159 cases out of 2,695), still increased to more than 95% in 2019 (10,636 cases out of 10,963). For other degenerative conditions (H), the proportion of procedures involving implants shifted from being the minority in 2001 (18%, 1,421 out of 7,885) to being the majority in 2019 (63%, 9,896 out of 15,580).

**Figure 6:**
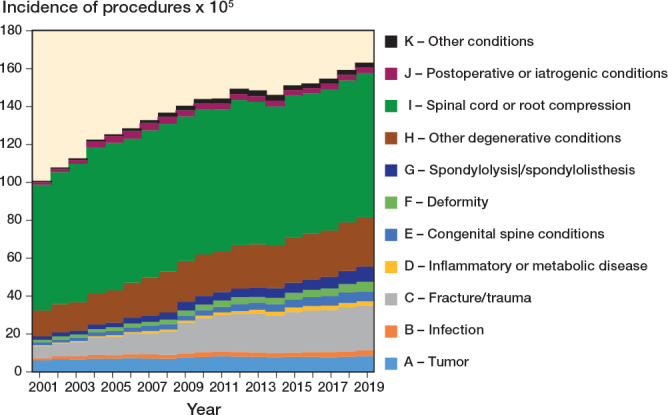
Time trends of incidence rates by diagnostic categories per 100,000 inhabitants (2001–2019).

**Figure 7:**
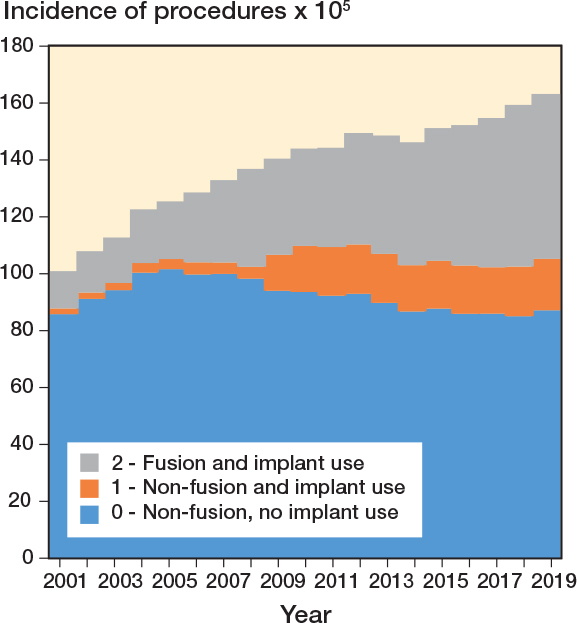
Time trends of incidence rates of procedures by procedure class per 100,000 inhabitants (2001–2019).

At the beginning of the study period, IR of procedures to treat spinal pathologies was 100.9 (CI 100.1–101.7) per 10^5^ inhabitants. In the following years, the rates increased to 163.2 (CI 162.2–164.3) per 10^5^ in 2019 (Table S4, see Supplementary data). The IR of procedures not involving fusion nor implant use remained stable from 85.8 (CI 85.0–86.5) in 2001 to 87.1 (CI 86.4–87.9) in 2019 with an IRR equal to 1 (CI 1–1). IRs of fusions with implants significantly increased in all the diagnostic categories according to IRRs (Table S4, see Supplementary data), but remained substantially stable for diagnostic category other conditions (K) when looking at the overall trend (P = 0.09). Considering diagnoses, the IR of all procedures increased significantly for all the diagnostic categories according to IRRs (Table S4, see Supplementary data), but showed no significant decreases for spinal cord or root compression (I) and postoperative or iatrogenic conditions (J) when looking at overall trends (P = 0.3). The incidence time trends over the 19 years considered showed an increase in case of fracture or trauma, inflammatory or metabolic disease, congenital spine conditions, deformity, spondylolysis or spondylolisthesis, and other degenerative conditions, for the diagnostic categories (Figure 6), and an increase in the procedures using implants, for the procedure classes (Figure 7).

## Discussion

This study aimed to explore trends in spinal surgery in Italy from 2001 to 2019, focusing on spinal implant usage in terms of surgical volumes and incidence at the population level. It also proposes a mapping from ICD9-CM codes to diagnostic categories and procedure classes to identify spinal pathologies and associated surgical treatments while using administrative data. To our knowledge, this is the first study on such a large scale in Italy involving the use of data from the HDR database of the Ministry of Health.

Over the observed 19 years, IR increased from 100.9 (CI 100.1–101.7) to 163.2 (CI 162.2–164.3) episodes per 10^5^ inhabitants. The trends showed important increases in implant procedures (+ 420%) to treat most diagnostic categories. The IRs increased overall due to several spine conditions.

Our study confirmed at a national level and on a broader period what Cortesi et al. observed in a single region in Italy concerning spinal fusions only [19]. At the international level, previous studies on the aggregated rates of spinal surgery come from Norway, Australia, the UK, Canada, and the USA. Grotle et al. [20] reported a significant increase in simple and complex lumbar spine surgery, mainly for fusion procedures, in Norway from 1999 to 2013, providing that the rate of lumbar spine surgery per 10^5^ inhabitants was estimated to increase by 54%, from 78 to 120. In Australia, spinal fusion procedures increased by 2% in the public and 167% in the private sector between 1997 and 2006, an even higher increase than that observed for hip or knee arthroplasties [21]. In England (UK), in a study on degenerative lumbar spine disease, Sivasubramaniam et al. reported that the number of procedures almost doubled between 1999 and 2013, with an incidence increasing from 24.5 to 48.8 per 10^5^ inhabitants [22]. In Ontario (Canada), an upward trend of lumbar fusion procedures was reported, increasing from 6.2 to 14.2 procedures per 100,000 between 1993 and 2012 [23]. In the United States, Sheikh et al. analyzed 7.1 million cases between 1998 and 2014, observing an increase of 118% in the number of spinal fusion procedures. Moreover, similar to our findings, they reported a decreasing trend in non-fusion spinal procedures [24].

We observed a change in the way Italian surgeons choose to treat several pathologies, with a dramatic increase in using implantable devices in case of trauma, congenital and degenerative conditions, and spondylolysis. These results reflected the findings of a study on trends in lumbar spine decompression and fusion surgery performed in Finland using administrative data from 1997 to 2018 [25]. Spinal cord and root compression are the most frequent diagnosis treated without implants. However, against a slight decrease in the number of such surgeries, the treatment of this diagnosis with implants increased more than 5-fold. In any case, all the surgeries without implants still represent the majority despite their overall slight decrease over time.

### Strengths

This study allows for a complete view of all the procedures performed in Italy, presenting data at the population level. The availability of official data from the Italian national HDR database, collecting population-based data over 19 years with a 94% to 99% coverage rate, makes the results representative of the Italian population and likely mirrors the situation of countries with similar populations and health systems. Moreover, this study provides a glance at the landscape of implant usage; the granularity of the data considered allows for accurate analyses of the trends and their implications in terms of healthcare impact and challenges, making the presented results an accurate picture of the reality for the entirety of a country’s healthcare. Our study is the first to provide nationwide longitudinal data for most types of spine surgery by treatment and diagnosis. Indeed, the retrieved published studies do not provide specific information on such a wide variety of spinal interventions, including both simple (e.g., microsurgical discectomy and/or decompression) and complex surgical procedures (e.g., deformity correction and treatment of tumors).

### Limitations

The proposed mapping is based on ICD9-CM coding, which may report limited or misleading information, as it is developed for administrative purposes. Moreover, we cannot be entirely sure about the clinical correctness of reported information, which can be evaluated only via audit at the national level. Indeed, because we used retrospective data from an administrative database, it was not possible to assess the correspondence between the reported and the actual diagnoses and procedures. Nonetheless, given the magnitude of our data, which provides information at the population level, the assumption of an existing national systematic error hardly would hold true.

One of the objectives of this study was to evaluate trends in large groups of diagnosis- and treatment-related episodes (such as fusion for degeneration, fusion for trauma, fusion for tumor, decompression for stenosis or disc herniation) and their related use of implants. The audits of the competent authority suggest that the coverage of HDR at the beginning of this study period was 94%, which in a few years, increased to 99%, and remained at this level until the end of the period observed. However, no specific year-to-year data or information on compliance in reporting specific procedures and diagnoses are available. Therefore, even though this is not expected to introduce significant alterations in numbers, it might still be a source of bias. Finally, since this study is based on only administrative data, it does not include information to detect relevant aspects of spinal surgery and describe the implanted devices. For instance, the impact of health status on the assignment of surgery, the baseline health status of patients, or the variations of health status following surgery, to cite some examples, have yet to be discovered. Moreover, though the authors are confident that the procedure classes defined in this study identify with fair precision surgeries where implants are used, the information contained in the administrative database did not allow for assessing the appropriateness of the indication for the procedures.

### Conclusion

We showed that the number of spinal surgical procedures in Italy and their population incidence rates increased significantly during the period observed. While non-implant-related procedures have maintained a relatively stable rate, fusions and other implant-related procedures have increased substantially for most diagnostic categories. All these changes may arguably be due to an enlargement of the treated population because of innovative technologies, such as new implantable devices, and improved surgical techniques that allow for treating more severe cases, and those previously untreated or treated by a conservative approach.

### Perspective

The high increase observed in the use of implants highlights the need to establish national registries with a high level of data completeness to monitor patients’ safety by ensuring medical device traceability and outcome assessment. Well-established and comprehensive registries would allow the collection of reliable data on adverse events, the monitoring of long-term safety and efficacy, and the quick traceability of patients in case of recall [26,27]. Following the need to apply to spinal implants the consolidated assessment approach used for joint replacements, the Orthopaedic Data Evaluation Panel (ODEP) (https://www.odep.org.uk/methodology/methodology-for-spine) in March 2023 launched an important worldwide initiative to allow cooperation among registries, to ensure the comparability of their findings, by defining standards to allow cross-border comparison of outcomes.

Moreover, we presented specific mapping based on ICD9-CM coding for spinal diagnoses and procedures as a reproducible tool for further explorations in our and other countries, possibly investigating additional factors associated with the rise in implant-related spine surgeries, such as sex, age, and hospital type.

We hope that this work might be a suitable reference for future studies that eventually might support decisions/policy-makers at the national and international levels.

### Supplementary data

Supplementary material is available in the online version of this article. Tables S1 and S2 (PDF) include the list of all the ICD9-CM codes selected for both diagnoses and procedures and their mapping into the a priori-defined diagnostic categories and procedures classes. Tables S3 and S4 (Excel file) comprise 2 sheets with 1 table each, reporting the total number of procedures performed (Table S3) and the IRs per 10^5^ inhabitants in Italy (Table S4). In both tables, information is reported by year and stratified by procedure class and diagnostic category. The supplementary data is available on the article page, doi: 10.2340/17453674.2025.43188

## Supplementary Material





## Notes

MT, AP, ECi, FL, GB, and PB: drafting of the manuscript; AP, FL, VV, GB, GZ, and PB: clinical expertise; ECa and VM: data extraction and data processing; ECi: data analysis and statistics; MT and PB design of the study; MT coordination of the project. All authors contributed to the interpretation and discussion of the results and content of the manuscript and approved the final version to be published.

This study was carried on within the project “Italian implantable prostheses registry (RIPI): realization of a platform integrating data flows for orthopedic prostheses, spinal devices, pacemakers and defibrillators, heart valves,” coordinated by the Italian National Institute of Health and supported by the General Directorate of Medical Devices and Pharmaceutical Service of the Italian Ministry of Health and within the “Project ECS 0000024 Rome Technopole – CUP B83C22002820006, NRP Mission 4 Component 2 Investment 1.5, Funded by the European Union – NextGenerationEU,” Partner Italian National Institute of Health, Spoke 2 (Technology transfer, new entrepreneurship, business incubation and acceleration) and Flagship project FP4 (Development, innovation and certification of medical and non-medical devices for health).

The authors would like to acknowledge Mascia Masciocchi, Attanasio Cornacchia, Paola Laricchiuta, and Paola Ciccarelli for their management support of the RIPI and RIDIS projects.

Handling co-editors: Paul Gerdhem and Robin Christensen

*Acta* thanks Antti Malmivaara and other anonymous reviewers for help with peer review of this manuscript.
