# Supplementary material for: Time trends in spine surgery in Italy: a nationwide, population-based study of 1,560,969 records of administrative health data from 2001 to 2019
Source: Acta Orthop. 2025 Mar 13;96:256–64. doi: 10.2340/17453674.2025.43188 (PMC11971841; doi:10.2340/17453674.2025.43188)
Supplement: Supplementary file 1 [file ActaO-96-43188-s1.pdf]

SUPPLEMENTARY MATERIAL

Table S1. List of selected ICD9-CM diagnosis codes by diagnostic category

Diagnostic Category	ICD9-CM	Description
A	1702	Malignant neoplasm of vertebral column, excluding sacrum and coccyx
A	1706	Malignant neoplasm of pelvic bones, sacrum, and coccyx
A	1922	Malignant neoplasm of spinal cord
A	1983	Secondary malignant neoplasm of brain and spinal cord
A	1985	Secondary malignant neoplasm of bone and bone marrow
A	2132	Benign neoplasm of vertebral column, excluding sacrum and coccyx
A	2136	Benign neoplasm of pelvic bones, sacrum, and coccyx
A	2253	Benign neoplasm of spinal cord
A	2375	Neoplasm of uncertain behavior of brain and spinal cord
A	2376	Neoplasm of uncertain behavior of meninges
A	2377	Neurofibromatosis
A	23770	Neurofibromatosis, unspecified
A	23771	Neurofibromatosis, type 1 [von recklinghausen's disease]
A	2380	Neoplasm of uncertain behavior of bone and articular cartilage
A	2392	Neoplasm of unspecified nature of bone, soft tissue, and skin
B	0134	Tuberculoma of spinal cord
B	01340	Tuberculoma of spinal cord, unspecified
B	01341	Tuberculoma of spinal cord, bacteriological or histological examination not done
B	01342	Tuberculoma of spinal cord, bacteriological or histological examination unknown (at present)
B	01343	Tuberculoma of spinal cord, tubercle bacilli found (in sputum) by microscopy
B	01344	Tuberculoma of spinal cord, tubercle bacilli not found (in sputum) by microscopy, but found by bacterial culture
B	01345	Tuberculoma of spinal cord, tubercle bacilli not found by bacteriological examination, but tuberculosis confirmed histologically
B	01346	Tuberculoma of spinal cord, tubercle bacilli not found by bacteriological or histological examination, but tuberculosis confirmed by other methods [inoculation of animals]
B	0135	Tuberculous abscess of spinal cord
B	01350	Tuberculous abscess of spinal cord, unspecified
B	01351	Tuberculous abscess of spinal cord, bacteriological or histological examination not done
B	01352	Tuberculous abscess of spinal cord, bacteriological or histological examination unknown (at present)
B	01353	Tuberculous abscess of spinal cord, tubercle bacilli found (in sputum) by microscopy
B	01354	Tuberculous abscess of spinal cord, tubercle bacilli not found (in sputum) by microscopy, but found by bacterial culture
B	01355	Tuberculous abscess of spinal cord, tubercle bacilli not found by bacteriological examination, but tuberculosis confirmed histologically
B	01356	Tuberculous abscess of spinal cord, tubercle bacilli not found by bacteriological or histological examination, but tuberculosis confirmed by other methods [inoculation of animals]

B	0150	Tuberculosis of vertebral column
B	01500	Tuberculosis of vertebral column, unspecified
B	01501	Tuberculosis of vertebral column, bacteriological or histological examination not done
B	01502	Tuberculosis of vertebral column, bacteriological or histological examination unknown (at present)
B	01503	Tuberculosis of vertebral column, tubercle bacilli found (in sputum) by microscopy
B	01504	Tuberculosis of vertebral column, tubercle bacilli not found (in sputum) by microscopy, but found by bacterial culture
B	01505	Tuberculosis of vertebral column, tubercle bacilli not found by bacteriological examination, but tuberculosis confirmed histologically
B	01506	Tuberculosis of vertebral column, tubercle bacilli not found by bacteriological or histological examination, but tuberculosis confirmed by other methods [inoculation of animals]
B	01570	Tuberculosis of other specified bone, unspecified
B	01571	Tuberculosis of other specified bone, bacteriological or histological examination not done
B	01572	Tuberculosis of other specified bone, bacteriological or histological examination unknown (at present)
B	01573	Tuberculosis of other specified bone, tubercle bacilli found (in sputum) by microscopy
B	01574	Tuberculosis of other specified bone, tubercle bacilli not found (in sputum) by microscopy, but found by bacterial culture
B	01575	Tuberculosis of other specified bone, tubercle bacilli not found by bacteriological examination, but tuberculosis confirmed histologically
B	01576	Tuberculosis of other specified bone, tubercle bacilli not found by bacteriological or histological examination, but tuberculosis confirmed by other methods [inoculation of animals]
B	3241	Intraspinal abscess
B	3249	Intracranial and intraspinal abscess of unspecified site
B	73005	Acute osteomyelitis, pelvic region and thigh
B	73008	Acute osteomyelitis, other specified sites
B	73015	Chronic osteomyelitis, pelvic region and thigh
B	73018	Chronic osteomyelitis, other specified sites
B	73025	Unspecified osteomyelitis, pelvic region and thigh
B	73028	Unspecified osteomyelitis, other specified sites
B	73078	Osteopathy resulting from poliomyelitis, other specified sites
B	73088	Other infections involving bone in diseases classified elsewhere, other specified sites
B	73095	Unspecified infection of bone, pelvic region and thigh
B	73098	Unspecified infection of bone, other specified sites
B	9966	
B	99660	Infection and inflammatory reaction due to unspecified device, implant, and graft
B	99666	Infection and inflammatory reaction due to internal joint prosthesis
B	99667	Infection and inflammatory reaction due to other internal orthopedic device, implant, and graft
C	7217	Traumatic spondylopathy
C	73313	Pathologic fracture of vertebrae

C	805	Fracture of vertebral column without mention of spinal cord injury
C	8050	Cervical, closed
C	80500	Closed fracture of cervical vertebra, unspecified level
C	80501	Closed fracture of first cervical vertebra
C	80502	Closed fracture of second cervical vertebra
C	80503	Closed fracture of third cervical vertebra
C	80504	Closed fracture of fourth cervical vertebra
C	80505	Closed fracture of fifth cervical vertebra
C	80506	Closed fracture of sixth cervical vertebra
C	80507	Closed fracture of seventh cervical vertebra
C	80508	Closed fracture of multiple cervical vertebrae
C	8051	Cervical, open
C	80510	Open fracture of cervical vertebra, unspecified level
C	80511	Open fracture of first cervical vertebra
C	80512	Open fracture of second cervical vertebra
C	80513	Open fracture of third cervical vertebra
C	80514	Open fracture of fourth cervical vertebra
C	80515	Open fracture of fifth cervical vertebra
C	80516	Open fracture of sixth cervical vertebra
C	80517	Open fracture of seventh cervical vertebra
C	80518	Open fracture of multiple cervical vertebrae
C	8052	Closed fracture of dorsal [thoracic] vertebra without mention of spinal cord injury
C	8053	Open fracture of dorsal [thoracic] vertebra without mention of spinal cord injury
C	8054	Closed fracture of lumbar vertebra without mention of spinal cord injury
C	8055	Open fracture of lumbar vertebra without mention of spinal cord injury
C	8056	Closed fracture of sacrum and coccyx without mention of spinal cord injury
C	8057	Open fracture of sacrum and coccyx without mention of spinal cord injury
C	8058	Closed fracture of unspecified vertebral column without mention of spinal cord injury
C	8059	Open fracture of unspecified vertebral column without mention of spinal cord injury
C	806	Fracture of vertebral column with spinal cord injury
C	8060	Cervical, closed
C	80600	Closed fracture of C1-C4 level with unspecified spinal cord injury
C	80601	Closed fracture of C1-C4 level with complete lesion of cord
C	80602	Closed fracture of C1-C4 level with anterior cord syndrome
C	80603	Closed fracture of C1-C4 level with central cord syndrome
C	80604	Closed fracture of C1-C4 level with other specified spinal cord injury
C	80605	Closed fracture of C5-C7 level with unspecified spinal cord injury
C	80606	Closed fracture of C5-C7 level with complete lesion of cord
C	80607	Closed fracture of C5-C7 level with anterior cord syndrome
C	80608	Closed fracture of C5-C7 level with central cord syndrome
C	80609	Closed fracture of C5-C7 level with other specified spinal cord injury
C	8061	Cervical, open
C	80610	Open fracture of C1-C4 level with unspecified spinal cord injury
C	80611	Open fracture of C1-C4 level with complete lesion of cord

C	80612	Open fracture of C1-C4 level with anterior cord syndrome
C	80613	Open fracture of C1-C4 level with central cord syndrome
C	80614	Open fracture of C1-C4 level with other specified spinal cord injury
C	80615	Open fracture of C5-C7 level with unspecified spinal cord injury
C	80616	Open fracture of C5-C7 level with complete lesion of cord
C	80617	Open fracture of C5-C7 level with anterior cord syndrome
C	80618	Open fracture of C5-C7 level with central cord syndrome
C	80619	Open fracture of C5-C7 level with other specified spinal cord injury
C	8062	Dorsal [thoracic], closed
C	80620	Closed fracture of T1-T6 level with unspecified spinal cord injury
C	80622	Closed fracture of T1-T6 level with anterior cord syndrome
C	80623	Closed fracture of T1-T6 level with central cord syndrome
C	80624	Closed fracture of T1-T6 level with other specified spinal cord injury
C	80625	Closed fracture of T7-T12 level with unspecified spinal cord injury
C	80627	Closed fracture of T7-T12 level with anterior cord syndrome
C	80628	Closed fracture of T7-T12 level with central cord syndrome
C	80629	Closed fracture of T7-T12 level with other specified spinal cord injury
C	8063	Dorsal [thoracic], open
C	80630	Open fracture of T1-T6 level with unspecified spinal cord injury
C	80631	Open fracture of T1-T6 level with complete lesion of cord
C	80632	Open fracture of T1-T6 level with anterior cord syndrome
C	80633	Open fracture of T1-T6 level with central cord syndrome
C	80634	Open fracture of T1-T6 level with other specified spinal cord injury
C	80635	Open fracture of T7-T12 level with unspecified spinal cord injury
C	80636	Open fracture of T7-T12 level with complete lesion of cord
C	80637	Open fracture of T7-T12 level with anterior cord syndrome
C	80638	Open fracture of T7-T12 level with central cord syndrome
C	80639	Open fracture of T7-T12 level with other specified spinal cord injury
C	8064	Closed fracture of lumbar spine with spinal cord injury
C	8065	Open fracture of lumbar spine with spinal cord injury
C	8066	Sacrum and coccyx, closed
C	80660	Closed fracture of sacrum and coccyx with unspecified spinal cord injury
C	80661	Closed fracture of sacrum and coccyx with complete cauda equina lesion
C	80662	Closed fracture of sacrum and coccyx with other cauda equina injury
C	80669	Closed fracture of sacrum and coccyx with other spinal cord injury
C	8067	Sacrum and coccyx, open
C	80670	Open fracture of sacrum and coccyx with unspecified spinal cord injury
C	80671	Open fracture of sacrum and coccyx with complete cauda equina lesion
C	80672	Open fracture of sacrum and coccyx with other cauda equina injury
C	80679	Open fracture of sacrum and coccyx with other spinal cord injury
C	8068	Closed fracture of unspecified vertebral column with spinal cord injury
C	8069	Open fracture of unspecified vertebral column with spinal cord injury
C	809	Ill-defined fractures of bones of trunk
C	8090	Fracture of bones of trunk, closed
C	8091	Fracture of bones of trunk, open
C	8390	Cervical vertebra, closed

C	83900	Closed dislocation, cervical vertebra, unspecified
C	83901	Closed dislocation, first cervical vertebra
C	83902	Closed dislocation, second cervical vertebra
C	83903	Closed dislocation, third cervical vertebra
C	83904	Closed dislocation, fourth cervical vertebra
C	83905	Closed dislocation, fifth cervical vertebra
C	83906	Closed dislocation, sixth cervical vertebra
C	83907	Closed dislocation, seventh cervical vertebra
C	83908	Closed dislocation, multiple cervical vertebrae
C	8391	Cervical vertebra, open
C	83910	Open dislocation, cervical vertebra, unspecified
C	83911	Open dislocation, first cervical vertebra
C	83912	Open dislocation, second cervical vertebra
C	83913	Open dislocation, third cervical vertebra
C	83914	Open dislocation, fourth cervical vertebra
C	83915	Open dislocation, fifth cervical vertebra
C	83916	Open dislocation, sixth cervical vertebra
C	83917	Open dislocation, seventh cervical vertebra
C	83918	Open dislocation, multiple cervical vertebrae
C	8392	Thoracic and lumbar vertebra, closed
C	83920	Closed dislocation, lumbar vertebra
C	83921	Closed dislocation, thoracic vertebra
C	8393	Thoracic and lumbar vertebra, open
C	83930	Open dislocation, lumbar vertebra
C	83931	Open dislocation, thoracic vertebra
C	8394	Other vertebra, closed
C	83940	Closed dislocation, vertebra, unspecified site
C	83942	Closed dislocation, sacrum
C	83949	Closed dislocation, vertebra, other
C	8395	Other vertebra, open
C	83950	Open dislocation, vertebra, unspecified site
C	83952	Open dislocation, sacrum
C	83959	Open dislocation, vertebra,other
C	846	Sprains and strains of sacroiliac region
C	8470	Sprain of neck
C	8471	Sprain of thoracic
C	8472	Sprain of lumbar
C	8473	Sprain of sacrum
C	9051	Late effect of fracture of spine and trunk without mention of spinal cord lesion
C	9520	Cervical
C	95200	C1-C4 level with unspecified spinal cord injury
C	95201	C1-C4 level with complete lesion of spinal cord
C	95202	C1-C4 level with anterior cord syndrome
C	95203	C1-C4 level with central cord syndrome
C	95204	C1-C4 level with other specified spinal cord injury
C	95205	C5-C7 level with unspecified spinal cord injury

C	95206	C5-C7 level with complete lesion of spinal cord
C	95207	C5-C7 level with anterior cord syndrome
C	95208	C5-C7 level with central cord syndrome
C	95209	C5-C7 level with other specified spinal cord injury
C	9521	Dorsal [thoracic]
C	95210	T1-T6 level with unspecified spinal cord injury
C	95211	T1-T6 level with complete lesion of spinal cord
C	95212	T1-T6 level with anterior cord syndrome
C	95213	T1-T6 level with central cord syndrome
C	95214	T1-T6 level with other specified spinal cord injury
C	95215	T7-T12 level with unspecified spinal cord injury
C	95216	T7-T12 level with complete lesion of spinal cord
C	95217	T7-T12 level with anterior cord syndrome
C	95218	T7-T12 level with central cord syndrome
C	95219	T7-T12 level with other specified spinal cord injury
C	9522	Lumbar spinal cord injury without evidence of spinal bone injury
C	9523	Sacral spinal cord injury without evidence of spinal bone injury
C	9591	Trunk
C	9598	Other specified sites, including multiple injury
C	V5417	Aftercare for healing traumatic fracture of vertebrae
C	V5427	Aftercare for healing pathologic fracture of vertebrae
D	720	Ankylosing spondylitis and other inflammatory spondylopathies
D	7200	Ankylosing spondylitis
D	7201	Spinal enthesopathy
D	7202	Sacroiliitis, not elsewhere classified
D	7208	Other inflammatory spondylopathies
D	72081	Inflammatory spondylopathies in diseases classified elsewhere
D	72089	Other inflammatory spondylopathies
D	7209	Unspecified inflammatory spondylopathy
D	731	Osteitis deformans and osteopathies associated with other disorders classified elsewhere
D	7310	Osteitis deformans without mention of bone tumor
D	7311	Osteitis deformans in diseases classified elsewhere
D	7312	Hypertrophic pulmonary osteoarthropathy
D	7318	Other bone involvement in diseases classified elsewhere
D	75651	Osteogenesis imperfecta
E	741	Spina bifida
E	7410	Spina bifida with hydrocephalus
E	74100	Spina bifida with hydrocephalus, unspecified region
E	74101	Spina bifida with hydrocephalus, cervical region
E	74102	Spina bifida with hydrocephalus, dorsal (thoracic) region
E	74103	Spina bifida with hydrocephalus, lumbar region
E	7419	Spina bifida without mention of hydrocephalus
E	74190	Spina bifida without mention of hydrocephalus, unspecified region
E	74191	Spina bifida without mention of hydrocephalus, cervical region
E	74192	Spina bifida without mention of hydrocephalus, dorsal (thoracic) region

E	74193	Spina bifida without mention of hydrocephalus, lumbar region
E	7542	Congenital musculoskeletal deformities of spine
E	7561	Anomaly of spine
E	75610	Anomaly of spine, unspecified
E	75612	Spondylolisthesis
E	75613	Absence of vertebra, congenital
E	75614	Hemivertebra
E	75615	Fusion of spine (vertebra), congenital
E	75616	Klippel-Feil syndrome
E	7562	Cervical rib
E	7569	Other and unspecified anomalies of musculoskeletal system
F	7320	Juvenile osteochondrosis of spine
F	7328	Other specified forms of osteochondropathy
F	737	Curvature of spine
F	7370	Adolescent postural kyphosis
F	7371	Kyphosis (acquired)
F	73710	Kyphosis (acquired) (postural)
F	73711	Kyphosis due to radiation
F	73712	Kyphosis, postlaminectomy
F	73719	Other kyphosis (acquired)
F	7372	Lordosis (acquired)
F	73720	Lordosis (acquired) (postural)
F	73721	Lordosis, postlaminectomy
F	73722	Other postsurgical lordosis
F	73729	Other lordosis (acquired)
F	7373	Kyphoscoliosis and scoliosis
F	73730	Scoliosis [and kyphoscoliosis], idiopathic
F	73731	Resolving infantile idiopathic scoliosis
F	73732	Progressive infantile idiopathic scoliosis
F	73733	Scoliosis due to radiation
F	73734	Thoracogenic scoliosis
F	73739	Other kyphoscoliosis and scoliosis
F	7374	Curvature of spine associated with other conditions
F	73740	Curvature of spine, unspecified, associated with other conditions
F	73741	Kyphosis associated with other conditions
F	73742	Lordosis associated with other conditions
F	73743	Scoliosis associated with other conditions
F	7378	Other curvatures of spine
F	7379	Unspecified curvature of spine
F	7385	Other acquired deformity of back or spine
F	7386	Acquired deformity of pelvis
G	7384	Acquired spondylolisthesis
G	75611	Spondylolysis, lumbosacral region
H	71858	Ankylosis of joint, other specified sites
H	721	Spondylosis and allied disorders
H	7210	Cervical spondylosis without myelopathy

H	7211	Cervical spondylosis with myelopathy
H	7212	Thoracic spondylosis without myelopathy
H	7213	Lumbosacral spondylosis without myelopathy
H	7214	Thoracic or lumbar spondylosis with myelopathy
H	72141	Spondylosis with myelopathy, thoracic region
H	72142	Spondylosis with myelopathy, lumbar region
H	7215	Kissing spine
H	7216	Ankylosing vertebral hyperostosis
H	7219	Spondylosis of unspecified site
H	72190	Spondylosis of unspecified site, without mention of myelopathy
H	72191	Spondylosis of unspecified site, with myelopathy
H	722	Intervertebral disc disorders
H	72231	Schmorl's nodes, thoracic region
H	72232	Schmorl's nodes, lumbar region
H	72239	Schmorl's nodes, other region
H	7224	Degeneration of cervical intervertebral disc
H	7225	Degeneration of thoracic or lumbar intervertebral disc
H	72251	Degeneration of thoracic or thoracolumbar intervertebral disc
H	72252	Degeneration of lumbar or lumbosacral intervertebral disc
H	7226	Degeneration of intervertebral disc, site unspecified
H	7227	Intervertebral disc disorder with myelopathy
H	72270	Intervertebral disc disorder with myelopathy, unspecified region
H	72271	Intervertebral disc disorder with myelopathy, cervical region
H	72272	Intervertebral disc disorder with myelopathy, thoracic region
H	72273	Intervertebral disc disorder with myelopathy, lumbar region
H	7228	Postlaminectomy syndrome
H	72280	Postlaminectomy syndrome, unspecified region
H	72281	Postlaminectomy syndrome, cervical region
H	72282	Postlaminectomy syndrome, thoracic region
H	72283	Postlaminectomy syndrome, lumbar region
H	7229	Other and unspecified disc disorder
H	72290	Other and unspecified disc disorder, unspecified region
H	72291	Other and unspecified disc disorder, cervical region
H	72292	Other and unspecified disc disorder, thoracic region
H	72293	Other and unspecified disc disorder, lumbar region
H	723	Other disorders of cervical region
H	7237	Ossification of posterior longitudinal ligament in cervical region
I	3351	Spinal muscular atrophy
I	33510	Spinal muscular atrophy, unspecified
I	336	Other diseases of spinal cord
I	3360	Syringomyelia and syringobulbia
I	3368	Other myelopathy
I	3369	Unspecified disease of spinal cord
I	34400	Quadriplegia, unspecified
I	34401	Quadriplegia, C1-C4, complete
I	34402	Quadriplegia, C1-C4, incomplete

I	34403	Quadriplegia, C5-C7, complete
I	34404	Quadriplegia, C5-C7, incomplete
I	34409	Other quadriplegia
I	3441	Paraplegia
I	3442	Diplegia of upper limbs
I	3490	Reaction to spinal or lumbar puncture
I	3532	Cervical root lesions, not elsewhere classified
I	3533	Thoracic root lesions, not elsewhere classified
I	3534	Lumbosacral root lesions, not elsewhere classified
I	7220	Displacement of cervical intervertebral disc without myelopathy
I	7221	Displacement of thoracic or lumbar intervertebral disc without myelopathy
I	72210	Displacement of lumbar intervertebral disc without myelopathy
I	72211	Displacement of thoracic intervertebral disc without myelopathy
I	7222	Displacement of intervertebral disc, site unspecified, without myelopathy
I	7230	Spinal stenosis in cervical region
I	7240	Spinal stenosis, other than cervical
I	72400	Spinal stenosis, unspecified region
I	72401	Spinal stenosis, thoracic region
I	72402	Spinal stenosis, lumbar region, without neurogenic claudication
I	72409	Spinal stenosis, other region
I	7425	Other specified anomalies of spinal cord
I	7429	Unspecified congenital anomaly of brain, spinal cord, and nervous system
I	7674	Injury to spine and spinal cord due to birth trauma
I	9072	Late effect of spinal cord injury
I	9073	Late effect of injury to nerve root(s), spinal plexus(es), and other nerves of trunk
I	952	Spinal cord injury without evidence of spinal bone injury
I	9524	Cauda equina spinal cord injury without evidence of spinal bone injury
I	9528	Multiple sites of spinal cord injury without evidence of spinal bone injury
I	9529	Unspecified site of spinal cord injury without evidence of spinal bone injury
I	953	Dorsal [thoracic]
I	9530	Injury to cervical nerve root
I	9531	Injury to dorsal nerve root
I	9532	Injury to lumbar nerve root
I	9533	Injury to sacral nerve root
I	9534	Injury to brachial plexus
I	9535	Injury to lumbosacral plexus
I	9538	Injury to multiple sites of nerve roots and spinal plexus
I	9539	Injury to unspecified site of nerve roots and spinal plexus
I	954	Injury to other nerve(s) of trunk, excluding shoulder and pelvic girdles
I	9540	Injury to cervical sympathetic nerve, excluding shoulder and pelvic girdles
I	9541	Injury to other sympathetic nerve, excluding shoulder and pelvic girdles
I	9548	Injury to other specified nerve(s) of trunk, excluding shoulder and pelvic girdles
I	9549	Injury to unspecified nerve of trunk, excluding shoulder and pelvic girdles
I	955	Injury to peripheral nerve(s) of shoulder girdle and upper limb
I	9550	Injury to axillary nerve
I	9551	Injury to median nerve

I	9552	Injury to ulnar nerve
I	9553	Injury to radial nerve
I	9554	Injury to musculocutaneous nerve
I	9555	Injury to cutaneous sensory nerve, upper limb
I	9556	Injury to digital nerve, upper limb
I	9557	Injury to other specified nerve(s) of shoulder girdle and upper limb
I	9558	Injury to multiple nerves of shoulder girdle and upper limb
I	9559	Injury to unspecified nerve of shoulder girdle and upper limb
I	956	Injury to peripheral nerve(s) of pelvic girdle and lower limb
I	9560	Injury to sciatic nerve
I	9561	Injury to femoral nerve
I	9562	Injury to posterior tibial nerve
I	9563	Injury to peroneal nerve
I	9564	Injury to cutaneous sensory nerve, lower limb
I	9565	Injury to other specified nerve(s) of pelvic girdle and lower limb
I	9568	Injury to multiple nerves of pelvic girdle and lower limb
I	9569	Injury to unspecified nerve of pelvic girdle and lower limb
J	9964	Mechanical complication of internal orthopedic device, implant, and graft
J	99640	Unspecified mechanical complication of internal orthopedic device, implant, and graft
J	99641	Mechanical loosening of prosthetic joint
J	99647	Other mechanical complication of prosthetic joint implant
J	99649	Other mechanical complication of other internal orthopedic device, implant, and graft
J	99677	Other complications due to internal joint prosthesis
J	99811	Hemorrhage complicating a procedure
J	99812	Hematoma complicating a procedure
J	99813	Seroma complicating a procedure
J	9982	Accidental puncture or laceration during a procedure, not elsewhere classified
J	9984	Foreign body accidentally left during a procedure
J	99881	Emphysema (subcutaneous) (surgical) resulting from procedure
J	99889	Other specified complications of procedures not elsewhere classified
J	9989	Unspecified complication of procedure, not elsewhere classified
J	V644	Closed surgical procedure converted to open procedure
J	E874	Mechanical failure of instrument or apparatus during procedure
K	4332	Occlusion and stenosis of vertebral artery
K	43320	Occlusion and stenosis of vertebral artery without mention of cerebral infarction
K	43321	Occlusion and stenosis of vertebral artery with cerebral infarction
K	4351	Vertebral artery syndrome
K	4353	Vertebrobasilar artery syndrome
K	44324	Dissection of vertebral artery
K	7218	Other allied disorders of spine
K	7231	Cervicalgia
K	7236	Panniculitis specified as affecting neck
K	7238	Other syndromes affecting cervical region
K	7239	Unspecified musculoskeletal disorders and symptoms referable to neck

K	7246	Disorders of sacrum
K	7313	Major osseous defects
K	7391	Nonallopathic lesions, cervical region
K	7392	Nonallopathic lesions, thoracic region
K	7393	Nonallopathic lesions, lumbar region
K	7394	Nonallopathic lesions, sacral region
K	V5302	Fitting and adjustment of neuropacemaker (brain) (peripheral nerve) (spinal cord)
K	V5309	Fitting and adjustment of other devices related to nervous system and special senses
K	V641	Surgical or other procedure not carried out because of contraindication
K	V642	Surgical or other procedure not carried out because of patient's decision

Table A.1.2. List of selected ICD9-CM procedure codes by procedure class

Class	ICD9-CM	Description
0	0211	Simple suture of dura mater of brain
0	0294	Insertion or replacement of skull tongs or halo traction device
0	0295	Removal of skull tongs or halo traction device
0	03	Operations on spinal cord and spinal canal structures
0	030	Exploration and decompression of spinal canal structures
0	0301	Removal of foreign body from spinal canal
0	0302	Reopening of laminectomy site
0	0309	Other exploration and decompression of spinal canal
0	031	Division of intraspinal nerve root
0	032	Chordotomy
0	0332	Biopsy of spinal cord or spinal meninges
0	034	Excision or destruction of lesion of spinal cord or spinal meninges
0	035	Plastic operations on spinal cord structures
0	0351	Repair of spinal meningocele
0	0352	Repair of spinal myelomeningocele
0	0359	Other repair and plastic operations on spinal cord structures
0	036	Lysis of adhesions of spinal cord and nerve roots
0	039	Other operations on spinal cord and spinal canal structures
0	0390	Insertion of catheter into spinal canal for infusion of therapeutic or palliative substances
0	0393	Implantation or replacement of spinal neurostimulator lead(s)
0	0394	Removal of spinal neurostimulator lead(s)
0	0395	Spinal blood patch
0	0396	Percutaneous denervation of facet
0	0399	Other operations on spinal cord and spinal canal structures
0	4131	Biopsy of bone marrow
0	771	Other incision of bone without division
0	7729	Wedge osteotomy, other bones
0	774	Biopsy of bone
0	7740	Biopsy of bone, unspecified site
0	7749	Biopsy of bone, other bones

0	786	Removal of implanted devices from bone
0	7869	Removal of implanted devices from bone, other bones
0	7929	Open reduction of fracture without internal fixation, other specified bone
0	797	Closed reduction of dislocation
0	7970	Closed reduction of dislocation of unspecified site
0	8039	Biopsy of joint structure, other specified sites
0	805	Excision, destruction and other repair of intervertebral disc
0	8050	Excision or destruction of intervertebral disc, unspecified
0	8051	Excision of intervertebral disc
0	8052	Intervertebral chemonucleolysis
0	8059	Other destruction of intervertebral disc
0	8199	Other operations on joint structures
0	8622	Excisional debridement of wound, infection, or burn
0	8628	Nonexcisional debridement of wound, infection or burn
1	0353	Repair of vertebral fracture
1	778	Other partial ostectomy
1	7780	Other partial ostectomy, unspecified site
1	7789	Other partial ostectomy, other bones
1	785	Internal fixation of bone without fracture reduction
1	7859	Internal fixation of bone without fracture reduction, other bones
1	7939	Open reduction of fracture with internal fixation, other specified bone
1	8099	Other excision of joint, other specified sites
1	8165	Percutaneous vertebroplasty
1	8166	Percutaneous vertebral augmentation
1	845	Implantation of other musculoskeletal devices and substances
1	8458	Implantation of interspineous process decompression device
1	8459	Insertion of other spinal devices
1	846	Replacement of spinal disc
1	8460	Insertion of spinal disc prosthesis, not otherwise specified
1	8461	Insertion of partial spinal disc prosthesis, cervical
1	8462	Insertion of total spinal disc prosthesis, cervical
1	8463	Insertion of spinal disc prosthesis, thoracic
1	8464	Insertion of partial spinal disc prosthesis, lumbosacral
1	8465	Insertion of total spinal disc prosthesis, lumbosacral
1	8466	Revision or replacement of artificial spinal disc prosthesis, cervical
1	8467	Revision or replacement of artificial spinal disc prosthesis, thoracic
1	8468	Revision or replacement of artificial spinal disc prosthesis, lumbosacral
1	8469	Revision or replacement of artificial spinal disc prosthesis, not otherwise specified
2	810	Spinal fusion
2	8100	Spinal fusion, not otherwise specified
2	8101	Atlas-axis spinal fusion
2	8102	Other cervical fusion of the anterior column, anterior technique
2	8103	Other cervical fusion of the posterior column, posterior technique
2	8104	Dorsal and dorsolumbar fusion of the anterior column, anterior technique
2	8105	Dorsal and dorsolumbar fusion of the posterior column, posterior technique
2	8106	Lumbar and lumbosacral fusion of the anterior column, anterior technique

2	8107	Lumbar and lumbosacral fusion of the posterior column, posterior technique
2	8108	Lumbar and lumbosacral fusion of the anterior column, posterior technique
2	8129	Arthrodesis of other specified joints
2	813	Refusion of spine
2	8130	Refusion of spine, not otherwise specified
2	8131	Refusion of atlas-axis spine
2	8132	Refusion of other cervical spine, anterior column, anterior technique
2	8133	Refusion of other cervical spine, posterior column, posterior technique
2	8134	Refusion of dorsal and dorsolumbar spine, anterior column, anterior technique
2	8135	Refusion of dorsal and dorsolumbar spine, posterior column, posterior technique
2	8136	Refusion of lumbar and lumbosacral spine, anterior column, anterior technique
2	8137	Refusion of lumbar and lumbosacral spine, posterior column, posterior technique
2	8138	Refusion of lumbar and lumbosacral spine, anterior column, posterior technique
2	8139	Refusion of spine, not elsewhere classified
2	8162	Fusion or refusion of 2-3 vertebrae
2	8163	Fusion or refusion of 4-8 vertebrae
2	8164	Fusion or refusion of 9 or more vertebrae
2	8451	Insertion of interbody spinal fusion device
